# Generation of ribosomal protein S1 mutants for improving of expression of difficult to translate mRNAs

**DOI:** 10.1007/s00253-025-13406-4

**Published:** 2025-01-23

**Authors:** Laura R. K. Niemelä, András Pásztor, Alexander D. Frey

**Affiliations:** Department of Bioproducts and Biosystems, Aalto University, Espoo, Finland

**Keywords:** Translation initiation, *Escherichia coli*, 5’-untranslated region, Ribosomal protein S1, Mutation library

## Abstract

**Abstract:**

Metagenomes present a source for novel enzymes, but under 1% of environmental microbes are cultivatable. Because of its useful properties, *Escherichia coli* has been used as a host organism in functional genomic screens. However, due to differing expression machineries in the expression host compared to the source organism of the DNA sequences, screening outcomes can be biased. Here, we focused on one of the limiting processes—translation initiation. To that end, we created an operon-like screening system in *E. coli* to select mutants of the ribosomal protein S1 with more relaxed sequence requirements for 5’-untranslated regions of mRNAs. We created two mutation libraries of the ribosomal protein S1, one covering domains 3 and 4 (D3-D4) and the second covering domains 3 to 5 (D3-D5). Most mutants from library D3-D4 proofed to be specific for a particular UTR sequence and improved only expression from a single construct. Only mutant 3 from library D3-D4 led to increased expression of four different reporters improving fluorescence levels by up to 21%. Mutants isolated from D3-D5 library led up to 90% higher expression compared to the control, though the mutants with highest improvements exhibited a specialist phenotype. The most promising mutant, mutant 4, exhibited a generalist phenotype and showed increased expression in all six reporter strains compared to the control. This could indicate the potential for a more promiscuous translation initiation of metagenomic sequences in *E. coli* although at the price of smaller increases compared to specialist mutants.

**Key points:**

*• An operon-like selection system allowed to isolate generalist and specialist S1 mutants.*

*• S1 mutants improved translation of mRNAs with 5'-UTRs from metagenomic sequences.*

*• Use of S1 mutants could increase coverage from metagenomic libraries in functional screens.*

## Introduction

Under 1% of existing microorganisms are cultivatable. Therefore, methods for efficient screening and exploitation are needed (Amann et al. 1995; Lorenz and Schleper 2002). Functional metagenomic screens are sequence-independent methods for analyzing the microbiome of a certain habitat at a certain time point. The purpose of functional metagenomics screens is to find new gene products and new or alternative enzyme functions without the need for sequence information as this will bias the results towards already known protein families (Steele et al. 2009). The functional metagenomics analysis is based on the construction of metagenomic libraries and their screening in the host organism of choice. One of the main challenges in functional metagenomics is the selection of suitable host organisms as there are differences between the hosts and the metagenomic-inserts expression machineries, which lead to challenges in transcription and translation from the metagenomic libraries (Uchiyama and Miyazaki 2009).

*Escherichia coli* is a well-established host organism in metagenomic screening experiments. It has a wide genetic engineering toolbox and expresses heterologous DNA more efficiently than other hosts. However, screened libraries often lack diversity as certain gene products remain unexpressed (Gabor et al. 2007; Culligan et al. 2014). It has been a point of interest to improve *E. coli* as a host organism by modifying translation initiation fidelity, with the aim of expressing heterologous DNA more efficiently. The results of Maar et al. (2008) and Bernstein et al. (2007) have been promising, suggesting that modifying ribosomal effectors in the translation initiation stage would be productive (Gualerzi and Pon 2015).

The ribosomal protein S1 is an essential factor for translation initiation in *E. coli*. It serves for an additional way of initiating translation; hence, *E. coli* does not solely depend on the Shine-Dalgarno- (SD) and anti-Shine-Dalgarno-sequences (aSD) and their cooperation for initiating translation (Gualerzi and Pon 2015). S1 protein is composed of six domains. The first two domains bind to the 30S subunit of the ribosome, and domains 3 to 5 bind to mRNA forming a binding platform (Aliprandi et al. 2008; Byrgazov et al. 2015). However, recently evidence has been found that domain 2 might have a supporting role in mRNA binding (D’Urso et al. 2023). The formation of the 30S pre-initiation complex, a complex formed by the 30S subunit binding to a single-stranded region on the mRNA with interacting initiation factors and aminoacylated initiator-tRNA, is the rate-limiting step in translation initiation. Ribosomal protein S1 may facilitate this step; hence, it could be a desirable effector for modifications with the aim of increasing expression of foreign sequences in *E. coli* (Milón et al. 2012; Gualerzi and Pon 2015). The mechanics in which ribosomal protein S1 facilitates translation initiation have been studied widely, for example, its chaperone activity and how it binds with one arm immediately upstream of the SD-region on the mRNA (Qu et al. 2012; Duval et al. 2013). The chaperone activity of S1, its ability to destabilize secondary structures (Qu et al. 2012; Duval et al. 2013), is of importance in initiating translation as the process is complicated by secondary structures in the translation initiation region of mRNA. A high GC content has been connected to secondary structure formation in the 5'-UTR and consequently to lower translation (Voges et al. 2004). However, S1 preferably interacts with A/U-rich regions (Hajnsdorf and Boni 2012).

In this work, we aimed to increase the translation initiation of metagenomic sequences in *E. coli* by modifying ribosomal protein S1 for a more relaxed specificity for the 5'-UTR. We created ribosomal protein S1 mutant libraries covering domains 3 and 4 and domains 3 to 5, respectively. Libraries were screened with an operon reporter carrying three different metagenomic 5'-UTRs linked to three different fluorescence protein-encoding regions. The selected mutants were compared to each other and the control in their capability to enhance translation of foreign sequences. Selected mutant proteins showed superiority over the wildtype protein in their capability to enhance translation on a spectrum of different 5'-UTR sequences.

## Materials and methods

### Strains, plasmid constructs, and oligonucleotides

*Escherichia coli* TOP10 (F- *mcrA* Δ*(mrr*-*hsdRMS*-*mcrBC*) φ80*lacZ*ΔM15 Δ*lacX*74 *nupG recA*1 *araD*139 Δ(*ara*-*leu*)7697 *galE*15 *galK*16 *rpsL*(Str^R^) *endA*1 λ^−^) and BL21 (DE3) (F^–^ *ompT gal dcm lon hsdS*_*B*_(*r*_*B*_^–^*m*_*B*_^–^) λ(DE3 [*lacI lacUV5*-*T7p07 ind1 sam7 nin5*]) [*malB*^+^]_K-12_(λ^S^)) were used for cloning and experimental work, respectively. All used plasmids and oligonucleotides are listed in Tables 1 and 2, respectively.

**Table 1:** List of plasmids

Name	Insert	Source
pACYCDuet-1		Novagen
pBAD/Myc-His A		Invitrogen
pGFPuv	GFPcycle 3	Clontech
pAP2	pACYCDuet-1 with UTR and RBS1 removed (between *lac*O1 and *Eco*RI)	This work
pAP3	pAP2 with GFPc3 with additional *Nhe*I	This work
pAP4	Wt *rpsA*, pBAD-Myc_His_A backbone	This work
pAP6	GFPc3 with UTR1, pACYCDuet-1 backbone	This work
pAP7	GFPc3 with UTR2, pACYCDuet-1 backbone	This work
pAP9	GFPc3 with UTR4, pACYCDuet-1 backbone	This work
pAP10	GFPc3 with UTR5, pACYCDuet-1 backbone	This work
pAP11	GFPc3 with UTR6, pACYCDuet-1 backbone	This work
pAP12	GFPc3 with UTR7, pACYCDuet-1 backbone	This work
pAP15	as pAP9, but MCS2 removed	This work
pAP16	UTR4 with GFPc3, UTR5 with mOrange, UTR6 with mApple, pACYCDuet-1 backbone	This work
pAP17	UTR5-mORGANGE, pACYCDuet-1 backbone	This work
pAP18	UTR6-mApple, pACYCDuet-1 backbone	This work

**Table 2:** List of oligonucleotides used in this study

Name	Sequence
ONAP_01	GCCACCATGGCCACTGAATCTTTTGCTCAACTC
ONAP_03	ATTGCTCGAGTTACTCGCCTTTAGCTGCTTTG
ONAP_05	GCCGAAGCTTTATTTGTAGAGCTCATCCATGC
ONAP_06	ATTAGACGCGTTGCGCGAGAAG
ONAP_10	GCCAGAATTCGGAATTGTTATCCGCTCAC
ONAP_17	AGGCATGGAAGTTAAAGGTATCG
ONAP_18	TTTCAGACCCAGGGAGATACGA
ONAP_21	GCGCGAATTCATGGCTAGCAAAGGAGAAGAACTTTTCAC
ONAP_24	GATGAGCTCTACAAATAAGGTACCAGACTCTTGATTTTAAAAAATTTTAAAG
ONAP_25	CACAGATCTCTACTTGTACAGCTCGTCCATG
ONAP_26	TACAAGTAGAGATCTGTGACAGAATCCTG
ONAP_27	CATTATGCGGCCGCATTACTTGTACAGCTCGTCCATG
ONAP_34	TTTAACGCCCAGGGAGATACGTTC
ONAP_35	TCGTGCCGTTATCGAATCCG
ON_obseq_3_fwd	TCTGCACCTGGAAGGCAAAG

### Construction of UTR-GPF reporter constructs

UTR-GFP reporter constructs were assembled in pACYCDuet-1 as described below. The original 5’ UTR and RBS1 region of the pACYCDuet-1 plasmid was replaced with a PCR-amplified fragment lacking 5’ UTR and the RBS1. A 539 bp fragment was amplified using primers ONAP_06 and ONAP_10 using pACYCDuet-1 as template. The PCR-amplified fragment and the original vector were digested with *Eco*RI and *Mlu*I, and the resulting fragments were ligated generating plasmid pAP2 that lacks the 5’ UTR between *lacO* and the original start codon. pAP3 was generated by PCR amplification of GFPcycle 3 using oligonucleotides ONAP_21 and ONAP_05 and plasmid pGFPuv (Clontech) as template and inserting the resulting PCR fragment into *Eco*RI and *Hin*dIII digested pAP2.

Cloning of the eight 5'-UTRs (Table 3) into pAP3 was done using the exonuclease and ligase-independent cloning (ELIC) (Koskela and Frey 2015). Synthetic DNA fragments were designed that encompassed the UTR DNA sequences flanked by 5′ and 3′ sequences identical with the integration site of the acceptor vector pAP3. *Eco*RI-linearized plasmid pAP3 was mixed with the synthetic DNAs, and the mixture was transformed into competent cells creating UTR-GFP reporter constructs generating plasmids pAP6, pAP7, and pAP9 to pAP14.

**Table 3:** List of 5'-UTRs used

UTR	Species	Target gene	Sequence	GC content (%)	Ensembl transcript
UTR1	*Methanococcoides burtonii* (DSM6242)	Adenylate kinase (adk)	TGGTCAGATCATTCTTCGGACAGGAATAATTAAATAATGGAGAT	34	ABE51049
UTR2	*Methanoculleus marisnigri* DSM1498	Glyceraldehyde-3-phosphate dehydrogenase (gap)	ATTTGCAAACCAGCAACCTTTATTAATTTTTGTTCTGTATTCCA	29.5	ABN58418
UTR4	*Methanosarcina acetivorans* (C2A)	Phosphoglycerate kinase 1 (pgk1)	GGAGAAGTTGCAGCAGCTTATATTCTAACGAGACTTGACGGGGG	50	AAM06047
UTR5	*Methanosarcina barkeri* (DSM804)	Serine hydroxymethyltransferase (gly1)	AGACTCTTGATTTTAAAAAATTTTAAAGGAATAGGTTACAGGTG	27.3	AAZ71240
UTR6	*Methanospirillum hungatei* (JF-1)	Enolase 1 (eno1)	GTGACAGAATCCTGCAGCCTTGCAAGCATTGGAGCATAAAAGGA	47.7	ABD40768
UTR7	*Thermoplasma volcanium* (GSS1)	Ribose-phosphate pyrophosphokinase (prs)	TGTTACTCATTCGGACAACTTTTTAATATCCTTGATCATACTG	32.6	BAB59339

The tricistronic reporter plasmid pAP16 was generated using Gibson assembly. First, the second MCS of pAP9 was removed by digestion with *Bgl*II and *Kpn*I, filling of the overhangs with T4 DNA polymerase and self-ligation creating plasmid pAP15. Two synthetic genes with custom-made 5'-UTRs (UTR5-mOrange and UTR6-mApple) were obtained from Eurofins Genomics and inserted into *Nde*I/*Avr*II sites of pACYCDuet1. UTR5-mOrange and UTR6-mApple sequences were amplified with primer pairs ONAP_24 and ONAP_25 and ONAP 26 and ONAP_27, respectively, and inserted into the *Hin*dIII digested vector pAP15 using Gibson assembly creating pAP16.

### Mutant library generation

The wildtype *rpsA* gene was amplified from purified *E. coli* K12 W3110 genomic DNA using oligonucleotides ONAP_01 and ONAP_03 and inserted into *Nco*I or *Xho*I restriction sites of pBAD/Myc-His-A generating pAP4. A stop codon at the end of *RpsA* gene was included ensuring expression of an untagged protein.

Two different *rpsA* mutant libraries encompassing either domains 3 and 4 (D3-4) or domains 3 to 5 (D3-5) of *rpsA*, respectively, were generated following a two-step protocol. In the first step, megaprimers were prepared according to the GeneMorph II EZClone Domain Mutagenesis Kit instructions (Agilent Technologies). For the mutant library D3-4, 469 bp long megaprimers were created. The megaprimers were generated using primers ONAP_17 and ONAP_18 and plasmid pAP4 as template. For the mutant library D3-5, 730 base pair megaprimers were created. The megaprimers were generated using primers ONAP_17 and ONAP_34 and plasmid pAP4 as template. The megaprimers were purified employing agarose gel electrophoresis and a PCR and gel purification kit (Macherey–Nagel Nucleospin Gel and PCR Clean-up).

The megaprimers were employed in the EZClone PCR assembly of mutated pAP4 plasmids according to manufacturers’ instructions. PCR products were digested by *Dpn*I and were purified using gel electrophoresis and DNA purification. The resulting libraries were named D3-D4 and D3-D5. A fraction of the libraries was transformed into *E. coli* TOP10 cells and plated on Luria–Bertani (LB)-agar with 100 mg/l ampicillin. Plasmids were isolated from random clones and sent for sequencing (Eurofins Genomics) for determination of the mutation frequencies.

### Mutant selection and screening

The screening procedures were performed using a Hamilton Microlab Star liquid handling workstation. All reporter constructs were transformed into *E*. *coli* BL21 (DE3) and chemically competent reporter strains were generated thereof. The mutation libraries were transformed into the reporter strains, and colonies were selected on Nunc Omnitrays prepared with LB agar with 100 mg/l ampicillin (Amp) and 34 mg/l chloramphenicol (Cm). After an overnight incubation, colonies were counted using the Hamilton Microlab Star liquid handling workstation.

Colony picking was conducted by the robot from one to maximally three plates to achieve the required number of colonies for inoculating pre-cultures. Maximally four 96 deep well plates were inoculated at a time, prefilled with 1 ml/well of liquid LB media with 100 mg/l ampicillin and 34 mg/l chloramphenicol. The plates were covered with Enzyscreen CR1396 covers and fixed into Enzyscreen CR1800 clamp system for incubation at 37 °C with 250 rpm shaking. The overnight cultures were utilized to inoculate gamma sterilized 96 well U Nunc plates prefilled with 178 µl of selective M9 minimal media (Amp and Cm) containing 20 g/l glucose with 20 μl saturated inoculum.

Plates were incubated 2 h at 37 °C and 250 rpm shaking. Two hours after inoculation, the cultures were induced with 2 µl of a stock solution containing 50 mM isopropyl β-D-1-thiogalactopyranoside (IPTG) and 200 g/l L-arabinose resulting in a final concentration of 0.5 mM of IPTG and 2.0 g/l of arabinose. After induction of cells, plates were incubated for 5 h at 37 °C and 250 rpm shaking. Finally, OD_600_ and fluorescence on the respective wavelength were determined. The plate reading was conducted, depending on the experiment, with a Biotek Synergy 2 plate reader (experiments with the GFP reporter) or a Biotek Cytation 3 plate reader (experiments with the operon reporter).

The hit selection was based on the Z-score of fluorescence values, which were corrected with the corresponding OD_600_ values. The plate average OD_600_-corrected fluorescence value was subtracted from a single clone’s OD_600_-corrected fluorescence value and divided by the corresponding plate’s standard deviation. For hits selected with the GFP reporter, the threshold Z-score for selection was set to 2.5. For the hits selected with the operon reporter plasmid, the threshold Z-score was set to 2.0. A clone was selected as a hit if at least two of the three measured fluorescent proteins showed Z-scores above 2 and the third score was not negative. Glycerol stocks (20% glycerol) were prepared from selected clones and stored at − 80 °C.

For a comparative screening, selected mutants were inoculated from glycerol stocks into pre-cultures with 5 ml LB media with 100 mg/l ampicillin and 34 mg/l chloramphenicol and grown overnight at 37 °C. From each mutant, four replicate cultures were made. The protocol used for the comparative screening was identical with the screening with the exception that the tests were done with manual pipetting.

For testing the functionality of a selected mutant with another 5'-UTR, plasmid DNA was isolated from selected mutants, treated with *Spe*I, and separated on agarose gels in order to remove the reporter plasmid from the sample. Intact, non-digested plasmid DNA was purified and transformed into *E*. *coli* BL21 (DE3) strains harboring the different GFP reporter constructs. Testing was conducted as described above.

### Sequence analysis and visualization of mutants

The mutants selected by the pAP16 operon reporter and screened in comparison to controls and each other were sent for sequencing to Eurofins Genomics. Standard protocol for plasmid DNA sequencing preparation was followed, and ONAP_35 primer was employed for the samples sent for sequencing to Eurofins Genomics. The sequencing results were verified by a second round of sequencing using the ON_obseq_3_fwd primer.

Clone manager was used for sequence analysis and alignment. UCSF Chimera package was used for visualizing domains of S1 mutants, which were selected based on the sequencing and screening results. Chimera is developed by the Resource for Biocomputing, Visualisation, and Informatics at the University of California, San Francisco, supported by NIH P41-GM103311 (Pettersen et al. 2004). The Dunbrack 2010 rotamer library was used for visualizing the mutants with UCSF Chimera (Shapovalov and Dunbrack 2011).

## Results

### Selection of 5’-untranslated regions for screening

In the initial screenings, we were interested in finding the most effective 5'-UTRs for selecting ribosomal protein S1 mutants. For this purpose, we selected 5'-UTRs from six species that control translation of highly expressed mRNA molecules (Table 3). GC content of those 5'-UTRs varied between 27.3 and 50%. We created GFP reporter constructs controlled by a metagenomic 5'-UTR, transformed a S1 mutant library (D3-D4) into those strains, and selected S1 mutants with improved translational efficiency towards this 5'-UTR. The selection criteria were based on the Z-score calculated from the OD_600_-corrected fluorescence values. The selection threshold was set to 2.5.

**Fig. 1:**
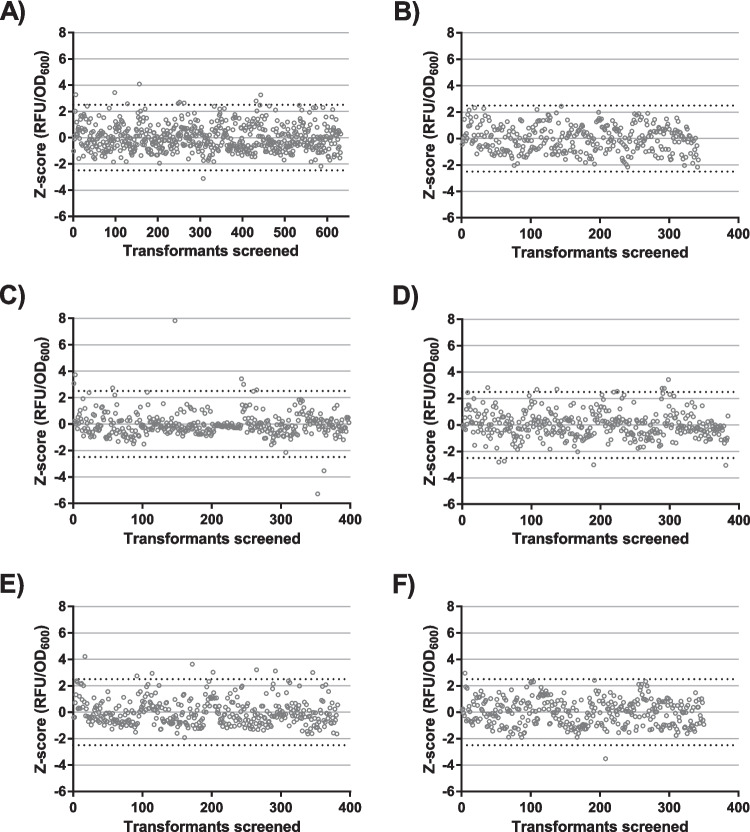
Selection of 5'-UTR sequences for efficient screening of S1 mutants. Six different GFP reporters, each controlled by a different metagenomic 5’-untranslated regions, were used to assess the efficiency to select S1 mutants from D3-D3 library. Hits were selected according to Z-scores based on OD_600_-corrected relative fluorescence (RFU/OD_600_). The threshold for hit selection was set at a Z-score of 2.5 and is marked with a dotted line. Circles depict screened transformants. **A** Using the GFP-UTR1, 9 out of 632 mutants met the selection criteria. **B** Using GFP-UTR2, 0 out of 343 mutants met the selection criteria. **C** Using GFP-UTR4, 7 out of 398 mutants met the selection criteria. **D** Using GFP-UTR5, 7 out of 384 mutants met the selection criteria. **E** Using GFP-UTR6, 8 out of 383 mutants were selected. **F** Using GFP-UTR7, 0 hits out of 342 mutants met the selection criteria

We screened 632 colonies with the UTR1 GFP reporter for improved translational efficiency and found nine hits (1.42% positive hits) (Fig. 1A). We screened 398 colonies with UTR4 and found 7 hits (1.76%), 384 colonies with UTR5 and found 7 hits (1.82%), and 383 colonies with UTR6 and found 8 colonies (2.09%) (Fig. 1C-E). We screened 342 colonies with a UTR2-controlled GFP reporter and 351 colonies with a UTR7-controlled GFP reporter without finding any hits meeting our selection criteria (Fig. 1B, F). Hence, mutants were selected only with GFP reporters controlled by UTR1, UTR4, UTR5, and UTR6, though the rate of finding positive hits varied to some extent. GC content of those UTRs varied between 27.3% or UTR5 and 50.0% for UR4, respectively. We chose UTR4, UTR5, and UTR6 for assembling an operon reporter construct as those 5'-UTRs led to the highest rates of finding positive hits.

### Screening of the D3-D4 library identified specialist mutants

Using the selected 5'-UTRs, an operon reporter plasmid (pAP16) was constructed. The operon reporter was constructed to select for S1 mutants with more relaxed sequence requirements of the UTRs and thus a more efficient translation initiation. Each 5'-UTR controlled the expression of a different fluorescence protein (UTR4-GFP, UTR5-mOrange, UTR6-mApple). The selection of the mutants was based on the Z-score calculated from the OD_600_-corrected fluorescence values. Compared to the single reporter system, we set a threshold of 2.0 for selecting hits.

**Fig. 2:**
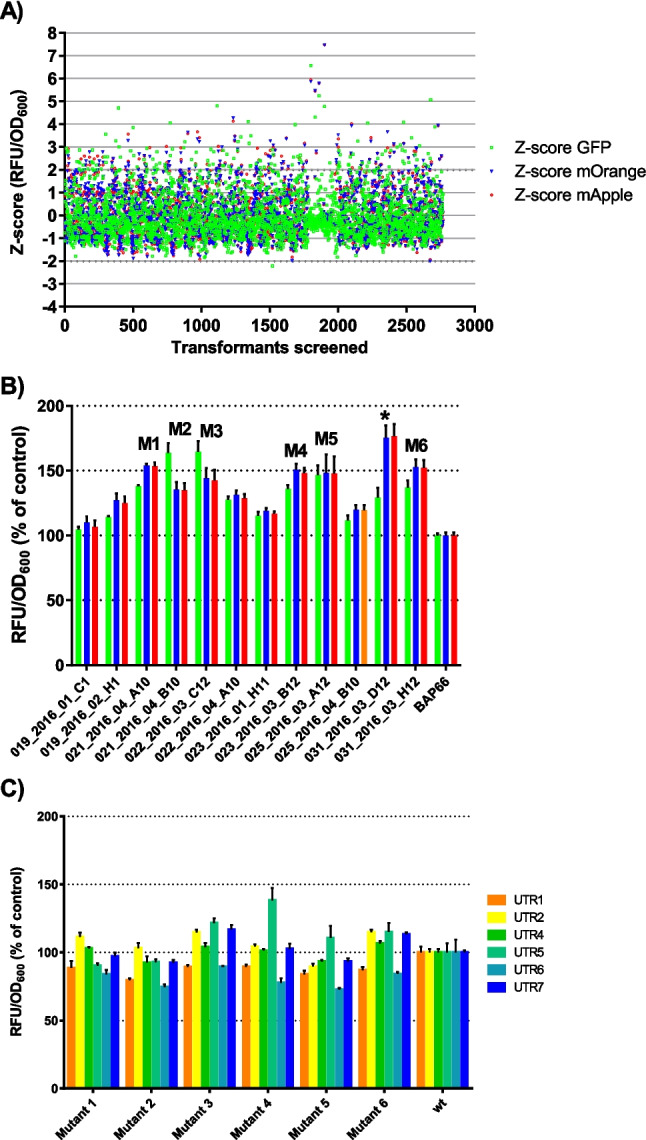
Selection and characterization of S1 mutants from D3-D4 library. **A** Selection of S1 mutants from D3-D4 library using the operon reporter. Hits were selected according to Z-scores based on OD_600_-corrected relative fluorescence (RFU/OD_600_). The green squares depict UTR4-controlled GFP expression, blue triangles UTR5-driven mOrange expression, and red circles UTR6-controlled mApple expression. Two out of the three reporters had to yield OD_600_-corrected relative fluorescence (RFU/OD_600_) over 2 and the third Z-score could not have a negative value. **B** Selected S1 mutants of mutation library D3-D4 in comparison to each other and to the control strain (wt S1 + pAP16). OD_600_-corrected relative fluorescence values (RFU/OD_600_) are reported as percentage of the control. The error bars indicate the standard deviation from four replicates. Color code of the reporters as in panel **A**. Mutants carried forward are marked M1 to M6; the mutant marked with an asterisk was excluded from further analysis. **C** Selected S1 mutants from the D3-D4 library were transformed into strains which contained the different GFP reporters. OD_600_-corrected relative fluorescence values (RFU/OD_600_) of the selected strains were compared to each other and the control and reported as percentage of control. The error bars indicate the standard deviation from four replicates

We screened about 2600 transformants of the mutation library D3-4 and selected 12 mutants. However, for 11 of these mutants, the selection criteria were only met by the UTR5-controlled mOrange and UTR6-controlled mApple fluorescence readouts (Fig. 2A). We conducted a follow-up screening to compare the 12 selected mutants with each other and the control. All clones expressing mutant ribosomal S1 proteins displayed 4.7–76.7% higher fluorescent protein expression compared to the control (Fig. 2B). The UTR4-controlled GFP expression in the mutants reached 104.7 to 164.4% of the control, while the UTR5-controlled mOrange and UTR6-controlled mApple expression reached 110.1 to 175.3% and 106.7 to 176.7%, respectively, compared to the control expressing the wildtype S1 protein.

We selected six of the best performing S1 mutant proteins and investigated if the mutant S1 proteins would be capable of enhancing translation initiation of a wider variety of sequences with higher efficiency than the wildtype S1 protein. One of the best performing mutants (031_2016_03_D12) was omitted from this test, due to strong differences in the enhancement of the fluorescence readouts for GFP and the remaining mOrange and mApple. For confirming the effects of the mutants, we expressed these mutants in the different reporter strains that we used for the selection of 5'-UTRs. The GFP expression levels ranged from 72.7 to 138.2% of levels obtained with the control (Fig. 2C). While some of the S1 mutants led to increased expression of GFP in several reporter strains, none of the mutants increased GFP levels in more than four reporter strains compared to the wildtype S1. Most improvements were observed when using UTR5-GFP and UTR4-GFP reporters, where 4, respectively 3, S1 mutants led to enhanced GFP levels. Encouragingly, also expression from 5'-UTRs that were not used in the selection of the mutants, UTR2 and UTR7, yielded improved GFP expression. Overall, the selected mutant proteins could increase expression of fluorescence proteins of the operon reporter; however, they failed to a large extent in improving expression when the 5'-UTR sequence context was changed. This indicated that instead of selecting promiscuous mutant proteins, S1 mutants specific for a reporter construct were selected.

### Screening of the D3-D5 library identified specialist and generalist mutants

**Fig. 3:**
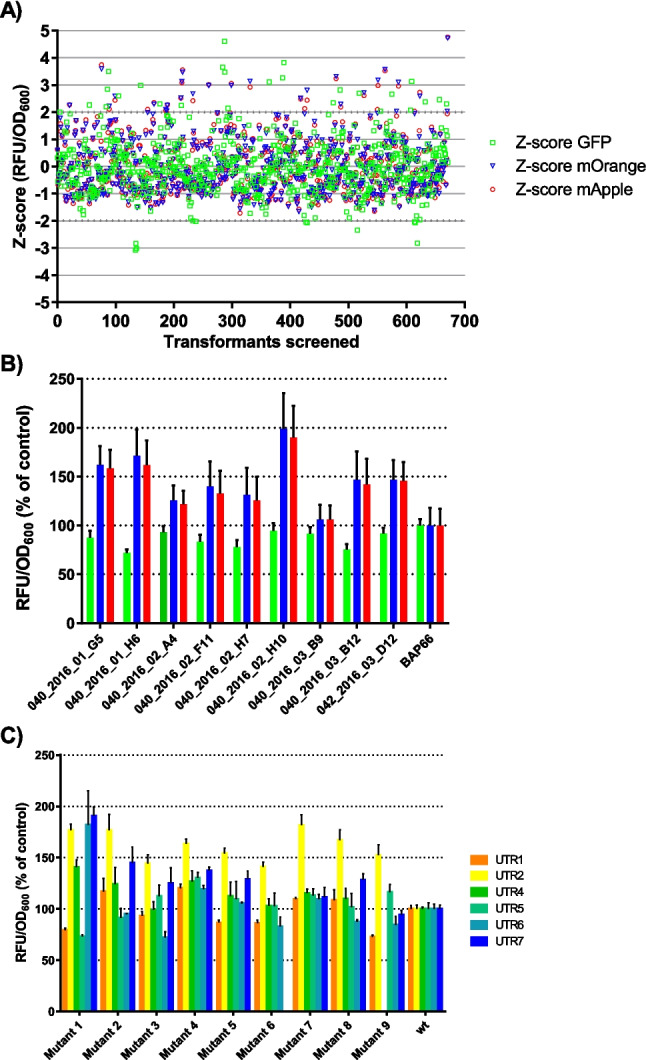
Selection and characterization of S1 mutants from D3-D5 library. **A** Selection of S1 mutants from D3-D5 library using the operon reporter. Hits were selected according to Z-scores based on OD_600_-corrected relative fluorescence (RFU/OD_600_). The green squares depict UTR4-controlled GFP expression, blue triangles UTR5-driven mOrange expression, and red circles UTR6-controlled mApple expression. Two out of the three reporters had to yield OD_600_-corrected relative fluorescence (RFU/OD_600_) over 2 and the third Z-score could not have a negative value. **B** Selected S1 mutants of mutation library D3-D5 in comparison to each other and to the control strain (wt S1 + pAP16). OD_600_-corrected relative fluorescence values (RFU/OD_600_) are reported as percentage of the control. The error bars indicate the standard deviation from four replicates. Color code of the reporters as in panel **A**. **C** Selected S1 mutants from the D3-D5 library were transformed into strains which contained the different GFP reporters. OD_600_-corrected relative fluorescence values (RFU/OD_600_) of the selected strains were compared to each other and the control and reported as percentage of control. The error bars indicate the standard deviation from four replicates. Two reporter strains did not grow and were eliminated from the results (UTR7 + mut6, UTR4 + mut9)

As domains 3 to 5 of S1 protein are crucial for mRNA binding, we created a new mutation library and extended the range for mutagenesis to cover domains 3 to 5 (library D3-D5). A total of 672 transformants from the mutation library D3-5 were screened with the pAP16 operon reporter, and from these, nine were selected as hits (Fig. 3A). We identified only partial hits, and the selection criteria were only met for UTR5-controlled mOrange fluorescence values and UTR6-controlled mApple values. We selected nine S1 mutants of the mutation library D3-D5, and these mutants were compared to each other and to the control strain for their efficiency of enhancing translation initiation. Expression of the UTR4-controlled GFP ranged from 71.9 to 94.6%, expression of UTR5-controlled mOrange from 106.1 to199.1%, and expression of UTR6-controlled mApple from 106.1 to 189.9% compared to the control (Fig. 3B).

Next, the nine selected S1 mutants were compared with each other and the wildtype employing six different GFP reporter plasmids (Fig. 3C). The objective was to test if the S1 mutants were able to initiate translation on various metagenomic 5'-UTRs in comparison to the corresponding control strain with an S1 wildtype and GFP reporter construct with the corresponding metagenomic 5'-UTR. In most mutants, the GFP sequences, which were controlled by UTR2 and UTR7, were highly expressed. The GFP reporter strains with S1 mutant 1, mutant 2, and mutant 4 showed the highest amount of expression, although standard deviations of the OD_600_-corrected fluorescence values (reported as percentage of average of control values) of some of these strains were quite high. The relative expression of the GFP reporters with the six different 5'-UTRs compared to the control ranged from 73.1 to 190.6% for mutant 1, 91.4 to 176.8% for mutant 2, and 119.2 to 163.5% for mutant 4. Notably, mutant 4 enhanced the expression from all the different GFP reporters compared to the control, yielding increases between 19.2 and 63.5%. In addition, the reporter strains with mutant 7 produced higher GFP expression in all reporter strains compared to the control, although most readouts were only slightly (9.5 to 15.4%) above the readouts of the control strains except for UTR2-controlled expression resulting in an 81.8% increase in GFP expression compared to the control.

### Sequence analysis of mutants selected from D3-D5 library

**Fig. 4:**
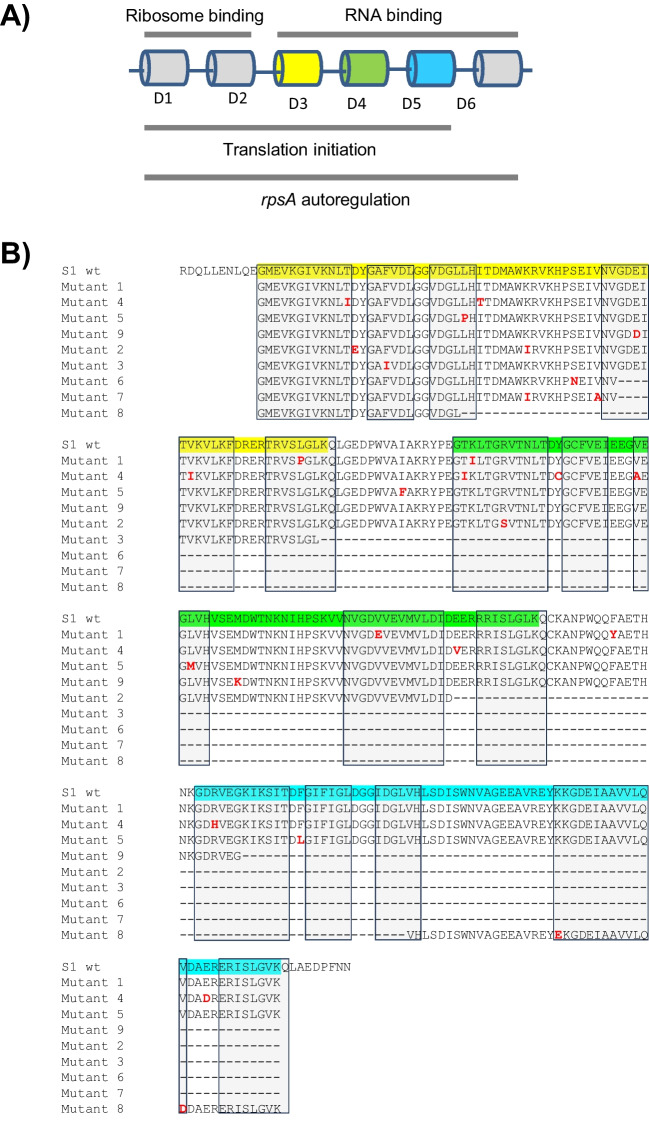
Comparison of mutants selected from D3-D5 library. **A** Schematic representation of the domain structure of S1 protein and their functions. The domain organization and some key functions of ribosomal protein S1. Domain 1 (D1) in position 21–87 and domain 2 (D2) in position 105–171 are involved in 30S binding. Domain 3 (D3) in position 192–260, domain 4 (D4) in position 277–347, domain 5 (D5) in position 364–434, and domain 6 (D6) in position 451–520 bind mRNA. Areas between the domains are flexible linkers (Byrgazov et al*.*, 2014; Duval et al. 2013). **B** The sequencing analysis of mutants of the mutation library OB3-5 and the wildtype is shown. Mutations are marked in red. Domains 3 to 5 are highlighted with color. The light grey boxes present the area of β-strands (Aliprandi et al. 2008; Salah et al., 2009)

The selected mutants were sequenced to analyze the changes in the sequence and the possible impact on the protein structure and function. The selected mutants contained between 2 and 12 mutations including missense, nonsense, and frameshift mutations, and one large deletion. Figure 4 depicts a cartoon of the domain architecture and the alignment of amino acids from domains 3 to 5 of wildtype S1 proteins and mutants selected from library D3-D5. From the mutation library D3-5, only three mutants produced a full-length protein being mutant 1, mutant 4, and mutant 5. Mutant 3, 6, and 7 contained mutations that led to truncation of the protein within or at the C-terminus of domain 3. Mutant 2 and mutant 9 were truncated in domain 4 and domain 5, respectively. Truncations occurred due to frameshift mutations, and two mutants were truncated as the mutations led to a premature stop codon. In mutant 8, we observed a rearrangement of the domains; domain 3 was found to be spliced to domain 5, leading to an S1 protein with 4 instead of 6 S1 motif domains (Fig. 4B).

We visualized the locations of the changes in the structures and analyzed the physicochemical properties of the mutants and estimated their impact on mRNA binding. We focused on four mutants, mutants 1, 2, and 7, whose changes seemed to lead to sequence preferences, as well as mutant 4, which exhibited the most interesting phenotypical effects. Mutant 4 also presented the highest number of changes in its sequence. From nine amino acid substitutions, seven were located in an area where the residues have been observed to participate in mRNA binding in the wildtype S1 protein (Aliprandi et al. 2008). Five of the amino acid substitutions led to significant changes in sidechain nature (hydrophobicity). Changes in sidechain nature (Kyte and Doolittle 1982) and participation of original residues to RNA binding according to previous studies (Aliprandi et al. 2008) are presented in Table 4.

**Table 4:** Summary of the effects of selected mutants selected from the ribosomal protein S1 mutation library D3-D5

Mutant	Amino acid substitutions	Original hydropathy index^a^	Replacing hydropathy index	Participation in mRNA binding^b^
Mutant 1	L257P	3.8	− 1.6	+
	K279I	− 3.9	4.5	-
	V327E	4.2	− 3.5	-
	F357Y	2.8	− 1.3	A specific interaction with some sequences
Mutant 2	D204E	− 3.5	− 3.5	A specific interaction with some sequences
	K226I	− 3.9	4.5	+
	R283S	− 4.5	− 0.8	-
	E337stop			A specific interaction with some sequences
Mutant 4	T203I	− 0.7	4.5	+
	I220T	4.5	− 0.7	A specific interaction with some sequences
	V243I	4.2	4.5	-
	T278I	− 0.7	4.5	+
	Y290C	− 1.3	2.5	+
	V300A	4.2	1.8	+
	E337V	− 3.5	4.2	A specific interaction with some sequences
	R366H	− 4.5	− 3.2	A specific interaction with some sequences
	E425D	− 3.5	− 3.5	A specific interaction with some sequences
Mutant 7	K226I	− 3.9	4.5	+
	V235A	4.2	4.5	+

^a^The change in hydrophobicity/hydrophilicity is presented with the hydropathy index (Kyte & Doolittle 1982)

^b^The participation of the site of amino acid substitution in mRNA binding is based on wildtype S1 protein and according to previous studies byAliprandi et al. (2008)

Sequence analysis indicated four amino acid substitutions in mutant 1. The amino acid substitution L257P was identified in domain 3 in the fifth β-sheet. The original amino acid residue in this location has been associated with mRNA binding. In domain 4, there were two amino acid substitutions, and both showed a change in hydrophobicity: K279I was located in the first β-sheet and V327E in the fourth β-sheet. The fourth amino acid substitution in mutant 1, F357Y, was in the linker between domain 4 and domain 5. This has also been previously identified as an RNA binding site. Mutant 2 was truncated as already noted, but it still proved to be one of the interesting mutants in the screening experiments. The first two amino acid substitutions were located in domain 3 in sites where RNA binding has been observed: D204E was located in the loop between the first and second β-sheet and K226I in the loop between the third and fourth β-sheet; the second amino acid substitution also caused a change in hydrophobicity. Two changes were located in domain 4: R283S and E337stop. The amino acid substitution R283S was located in the first β-sheet, and in the loop between the fourth and fifth β-sheet, a premature stop codon led to truncation (E337stop). This is a location which has been observed as an RNA binding site. Sequence analysis revealed that mutant 7 was truncated due to frameshift mutations in domain 3 in the fourth β-sheet. It presented two amino acid substitutions in the truncated third domain: K226I in the loop between the third and fourth domain and V235A just before the β-sheet. In both locations, the original residues have been observed to participate in RNA binding, and the amino acid substitution K226I also led to a change in hydrophobicity.

### Structural changes observed in the generalist mutant 4

We conducted a more thorough analysis of the effects of the different mutations of mutant 4 on the protein structure. The following changes were observed in mutant 4 considering the sequence and structure. In domain 3, the first two mutation locations have been associated with mRNA binding (Aliprandi et al. 2008): mutation T203I is located at the first β-sheet end and I220T at the loop between the third and fourth β-sheet. Both mutations also showed a change in hydrophobicity. The third mutation in domain 3, V243I, is located in the fourth β-sheet. The mutations in domain 4 are all located in sites which have been associated with mRNA binding: mutation T2781 is located in the first β-sheet, linker to the β-sheet region; it also showed a change in sidechain nature. Mutation Y290C is found in the linker between the first and second β-sheet and indicates a change in hydrophobicity. Mutation V330A is located in the third β-sheet, and mutation E337V is in the loop between the fourth and fifth β-sheet and shows a change in sidechain nature. Both mutations occurring in domain 5 are located in sites which have been linked to mRNA binding: mutation R366H is located in the first β-sheet and mutation E425D in the linker between the fourth and fifth β-sheet.

**Fig. 5:**
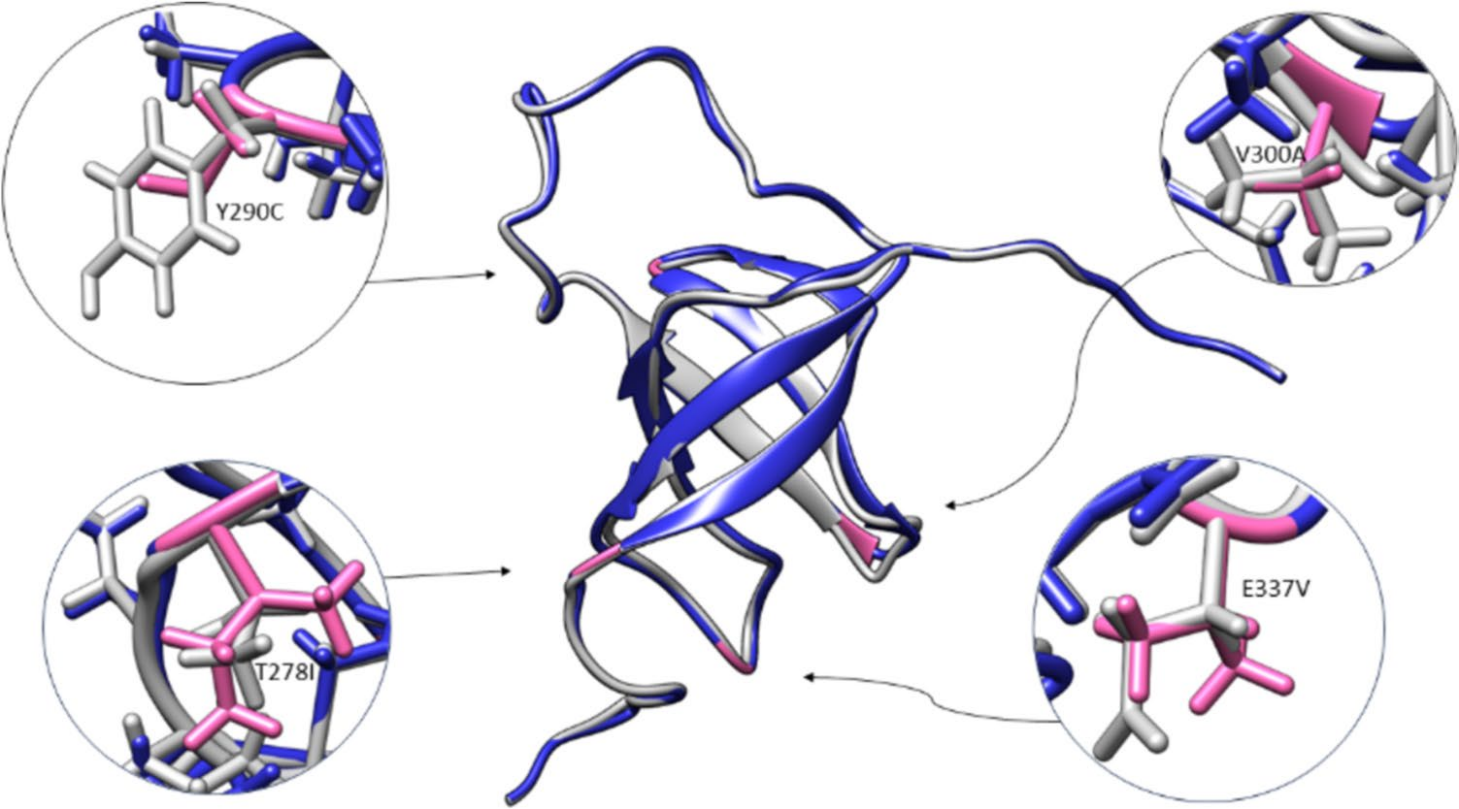
Modeling effect of amino acid substitutions on structure. Domain 4 of mutant 4 of the mutation library OB3-5 is displayed as a molecular model and compared to the wildtype domain 4. The wildtype is colored light grey and the mutant is colored blue. The four locations where mutations have led to amino acid changes (T278I, Y290, V300A, and E337V) have been highlighted pink. (Data for wildtype S1 by Salah et al., 2009; UCSF Chimera was used to create the visualization)

Figure 5 displays a molecular model of mutant 4 domain 4 in an overlay with the wildtype, highlighting the locations amino acid changes have occurred due to mutations and the possible orientations of the sidechains. The mutant domain visualization is based on the wildtype structure data by Salah et al. (2009). The Dunbrack 2010 rotamer library was used for visualizing the mutant domain 4 with UCSF Chimera (Shapovalov and Dunbrack 2011).

## Discussion

The aim of this work was to improve the translational process of mRNA sequences in *E. coli* by creating ribosomal protein S1 mutants with improved capability of binding and unfolding mRNA. Such mutants could be applied to improve expression of metagenomic libraries. Two mutation libraries of ribosomal protein S1 were constructed. The initial mutation library covered the S1 domains 3–4. A second mutation library of ribosomal protein S1 was constructed by expanding the area of induced mutations to domains 3–5 with the objective to improve results. Four promising mutants were found by screening the mutation library D3-D5.

Despite screening a much smaller number of colonies, the results with mutation library D3-D5 seemed more promising than with the mutation library D3-D4. One difference between the two libraries was the mutation rate. We sequenced random colonies to determine the mutation rates, for the D3-D4 library. The mutation rate was 10.5 mutations per kb. For clones of the D3-D5 library, the average mutation rate was 6.8 mutations per kb.

Another key difference was that the region for mutations was extended into the D5 region. This might be a consequence of the structure and function of the S1 mRNA binding platform. Aliprandi et al. (2008) described all these domains as mRNA binding domains but showed a difference in how they are associated with each other and mRNA. Qu et al. (2012) suggested a model for unwinding and rezipping RNA structures by S1. According to this model, binding RNA by S1 domains 4–5 is rate limiting for unwinding. This might also shed light on why expanding the area of the mutation library to domain 5 improves the results. However, from the S1 mutants with phenotypical interesting changes, only mutant 4 has a single amino acid substitution in D5, and mutant 2 and mutant 7 were missing this domain entirely.

The six final mutants of mutation library D3-D4 presented higher expression of UTR2-, UTR5-, and UTR7-controlled GFP. The strains with UTR4-controlled GFP presented mostly lower expression values. These mutants were selected mostly by UTR5-controlled mOrange expression and UTR6-controlled mApple values during hit picking. Also, these mutants seemed to present overall GC content dependency as GFP expression was highest when controlled by 5'-UTRs with a low GC content. Hence, these mutants were not showing the potential as generalists in initiating translation.

Generally, mutants 1 and 2 of the mutation library D3-D5 showed the highest protein expression values in the comparison and confirmation screenings. At the same time, the sequence of mutant 2 was truncated in domain 4. In previous studies, it has been observed that if ribosomal protein S1 is truncated and domains 1–4 remain, transcriptional cycling is impaired, but strains have still been observed to grow (Duval et al. 2013). The sequence of mutant 2 was truncated at the end of the fourth domain at a position which has been observed to participate in the binding of some mRNA sequences (Aliprandi et al. 2008). This could explain why the reporter strain expressing this mutant displayed mediocre to promising expression values and was not diminished in growth. Mutant 7 presented somewhat unexpected results. According to the sequencing results, this mutant carries a deletion in its third domain leading to frameshift and truncation which does not bode well for its function. Still, screening results with this mutant seemed promising as all reporter strains with mutant 7 showed higher expression values than the control strains in confirmation screenings.

All reporter strains with mutant 4 showed stably higher expression values than the control strains in confirmation screenings, which makes it the most versatile and hence the most interesting mutant. The selected S1 protein mutant 4 contained nine amino acid substitutions due to mutations, eight of these were overlapping with locations which had previously been identified by Aliprandi et al. (2008) as mRNA binding sites: four at a location for a more general type of binding and four at sites for binding more specifically mRNA. Five of these mutations displayed changes in sidechain nature; ergo, there was a change in hydrophobicity from the original to the replacing amino acid residue.

Bernstein et al. (2007) found that by doing multiple rounds of mutations with directed evolution, a lesser dependence on the GC content of the sequence was achieved. Similarly, in our work with mutant 4 as GFP expression seemed constantly increased from all the sequences which were controlled by the different 5'-UTRs (UTR1-7), the effect of the GC content was not seen as clearly as with the other mutants. This could indicate a more relaxed translation initiation of foreign sequences due to mutations in areas where mRNA binding has been observed to occur.

It is hard to predict the actual structural changes the mutations will induce on the protein especially as it exists in an unbound and bound state. How would the structural changes affect the function of the S1 protein? Our interest is focused on the mRNA binding platform which is formed by domains 3 to 5. Hence, the question would be if the mutations would influence the organization of the mRNA binding platform. Our visualization of domain 4 from mutant 4 domain 4 (Fig. 5) superimposed with the same domain of the wildtype protein has the purpose of making the case more tangible, but at the same time, set visualization is highly speculative. It shows possible orientations of the sidechains in mutation locations without any consideration on the other domains and their location. In this respect, the research of Qureshi et al. provides a more complete picture of the structure and molecular interactions. In their work, the structure of domain 3 and domain 4 of the ribosomal S1 protein from *Vibrio vulnificus* was resolved by NMR. Furthermore, structural insights on the dynamic interactions of the protein with an RNA molecule were reported (Qureshi et al. 2021).

Alternative methods for hit picking were discussed for achieving a broader selection of ribosomal protein S1 mutants, which could be employed for a more relaxed translation initiation. Instead of mutants in strains, which give high protein expression values, mutants with mediocre expression values could be selected for a broader range of translation initiation. Using an *E. coli*–based cell-free system as a base for screening would also be interesting. Sheahan and Wieden (2022) increased protein yield by increasing translation initiation in a cell-free system by adding purified ribosomal protein S1 into the system. It would be interesting to test an *E*. *coli*–based cell-free system for screening purposes with our S1 protein mutant. Moreover, S1 proteins are of importance for full activation of riboswitches (de Jesus et al. 2021); thus, modified S1 proteins might be useful tools to further modulate the activity of such switches.

Overall, the approach was successful in identifying ribosomal protein S1 mutants that have the potential to improve translation of mRNA sequences with a wide range of 5’ UTRs. While such mutants are of no interest when expressing a small number of genes, and thus the 5'-UTRs can be exchanged from improved expression, such mutants are of interest when performing functional genomics.

## Data Availability

All data pertaining to the findings of this study are available within this paper. Unprocessed raw data is provided upon request. All materials are shared with academic institutions upon request and execution of a material transfer agreement.
